# Extracellular vesicles released by microglia and macrophages carry endocannabinoids which foster oligodendrocyte differentiation

**DOI:** 10.3389/fimmu.2024.1331210

**Published:** 2024-02-23

**Authors:** Marta Lombardi, Federica Scaroni, Martina Gabrielli, Stefano Raffaele, Elisabetta Bonfanti, Fabia Filipello, Paola Giussani, Silvia Picciolini, Nicole Kerlero de Rosbo, Antonio Uccelli, Maria Teresa Golia, Giulia D’Arrigo, Tiziana Rubino, Kourosh Hooshmand, Cristina Legido-Quigley, Chiara Fenoglio, Alice Gualerzi, Marta Fumagalli, Claudia Verderio

**Affiliations:** ^1^ Department of Biomedical Sciences, National Research Council (CNR) Institute of Neuroscience, Vedano al Lambro, Italy; ^2^ NeuroMI Milan Center for Neuroscience, University of Milano-Bicocca, Milan, Italy; ^3^ Department of Pharmacological and Biomolecular Sciences “Rodolfo Paoletti”, Università degli Studi di Milano, Milan, Italy; ^4^ Scientific Institute for Research, Hospitalization and Healthcare (IRCCS) Humanitas Research Hospital, Rozzano, Italy; ^5^ Department of Medical Biotechnology and Translational Medicine, Università degli Studi di Milano, Segrate, Italy; ^6^ Scientific Institute for Research, Hospitalization and Healthcare (IRCCS) Fondazione Don Carlo Gnocchi Onlus, Milan, Italy; ^7^ Scientific Institute for Research, Hospitalization and Healthcare (IRCCS) Ospedale Policlinico San Martino, Genoa, Italy; ^8^ TomaLab, Institute of Nanotechnology, CNR, Rome, Italy; ^9^ Department of Neurology, Rehabilitation, Ophthalmology, Genetics, Maternal and Child Health (DINOGMI), University of Genoa, Genoa, Italy; ^10^ Department of Biotechnology and Life Sciences (DBSV) and Neuroscience Center, University of Insubria, Busto Arsizio, Italy; ^11^ System Medicine, Steno Diabetes Center Copenhagen, Copenhagen, Denmark; ^12^ Institute of Pharmaceutical Sciences, Faculty of Life Sciences and Medicine, King’s College London, London, United Kingdom; ^13^ Department of Biomedical, Surgical and Dental Sciences, Università degli Studi di Milano, Milan, Italy; ^14^ Fondazione Scientific Institute for Research, Hospitalization and Healthcare (IRCCS) Ca’ Granda, Ospedale Maggiore Policlinico, Milan, Italy

**Keywords:** macrophages, microglia, extracellular vesicles, oligodendrocytes, endocannabinoids, anandamide, 2-arachidonoylglycerol

## Abstract

**Introduction:**

Microglia and macrophages can influence the evolution of myelin lesions through the production of extracellular vesicles (EVs). While microglial EVs promote in vitro differentiation of oligodendrocyte precursor cells (OPCs), whether EVs derived from macrophages aid or limit OPC maturation is unknown.

**Methods:**

Immunofluorescence analysis for the myelin protein MBP was employed to evaluate the impact of EVs from primary rat macrophages on cultured OPC differentiation. Raman spectroscopy and liquid chromatography-mass spectrometry was used to define the promyelinating lipid components of myelin EVs obtained *in vitro* and isolated from human plasma.

**Results and discussion:**

Here we show that macrophage-derived EVs do not promote OPC differentiation, and those released from macrophages polarized towards an inflammatory state inhibit OPC maturation. However, their lipid cargo promotes OPC maturation in a similar manner to microglial EVs. We identify the promyelinating endocannabinoids anandamide and 2-arachidonoylglycerol in EVs released by both macrophages and microglia in vitro and circulating in human plasma. Analysis of OPC differentiation in the presence of the endocannabinoid receptor antagonists SR141716A and AM630 reveals a key role of vesicular endocannabinoids in OPC maturation. From this study, EV-associated endocannabinoids emerge as important mediators in microglia/macrophage-oligodendrocyte crosstalk, which may be exploited to enhance myelin repair.

## 1 Introduction

Oligodendrocytes (OLs) support fast nerve impulse transmission in the brain by insulating axons with myelin, which enwraps and protects axons. Loss of myelin in neuroinflammatory diseases, including multiple sclerosis (MS), results in impaired transmission, axonal damage, and neurological symptoms in affected patients (1–3).

Upon myelin damage, oligodendrocyte precursor cells (OPCs) migrate to the myelin lesion, undergo cycles of proliferation and eventually differentiate to generate new myelin-forming OLs enwrapping denuded axons (1, 4). However, spontaneous myelin repair eventually fails, especially in progressive forms of MS, due to an unfavorable persisting inflammatory environment that impairs successful maturation of OPCs, blocking them into an immature stage (5). Thus, the development of therapeutic approaches promoting remyelination is an unmet need in MS.

Myelin is a membrane structure characterized by extremely high lipid content (about 80% of dry weight) (2, 6, 7). Fatty acids are fundamental constituents for both phospholipids and glycolipids, abundant lipid myelin components, besides cholesterol (8). Fatty acids can be synthetized in OLs or can be provided to OLs by adjacent cells, which take them up from blood following dietary intake (9–11).

Extracellular vesicles (EVs) have recently emerged as lipid delivery vehicles between cells (12). EVs are small structures enveloped by a lipid bilayer that play key role in intercellular communication by the transfer of bioactive cargoes. EVs are lipids enriched, with a lipid over protein weight ratio that is about 8-fold higher in EVs compared to parent cells. Sphingomyelin, glycosphingolipids, serine phospholipids and cholesterol are among the most enriched lipid species in EVs (12, 13).

We previously found that microglia, the brain-resident immune cells, deliver promyelinating lipid(s) to OPCs through EVs, boosting OPC differentiation and myelin regeneration in a murine model of focal myelin lesion (14). However, the pro-myelinating lipid(s) of microglial EVs have not yet been identified. In addition, it is currently unclear whether pro-myelinating lipids may act as substrates (building blocks) for myelin synthesis or as signaling mediators that regulate oligodendrocyte response during myelin repair. Consistent with the latter possibility, previous evidence has indicated that microglia-derived EVs carry a lipid messenger, the endocannabinoid anandamide (AEA) (15), which, through cannabinoid (CB)1 receptor activation, may drive OPC differentiation into mature OLs (16, 17).

In this study, we sought to identify pro-myelinating lipid(s) in microglial EVs by a differential spectroscopy analysis, using EVs unable to support OPC differentiation as reference samples. To this purpose, we first explored whether EVs produced by bone-marrow-derived macrophages, the peripheral counterpart of microglia, may be unable to support OPC differentiation and thus be used to identify differentially expressed lipids in microglial EVs. Indeed, together with microglia, infiltrating macrophages are present in active demyelinating lesions of the human brain and spinal cord (18) and often play opposite functions in neuroinflammatory diseases (19). We found that macrophage-derived EVs did not promote differentiation of cultured OPCs, but unexpectedly their lipid cargo enhanced OPC maturation in similar manner to that of microglial EVs. Hence, we took advantage of Raman spectroscopy and Ultra High-Performance Liquid Chromatography (UHPLC) to identify common (instead of differentially expressed) pro-myelinating lipids in EVs derived from cultured cells and validated their presence in human blood myeloid EVs.

## 2 Materials and methods

### 2.1 Primary cultures

All animal procedures were carried out according to the European directive (2010/63/EU), the Italian Law for Care and Use of Experimental Animals (DL116/92; DL26/2014), and were approved by the Italian Ministry of Health (Authorization: 1112-2016PR).

#### 2.1.1 Bone-marrow-derived macrophages

**Figure 1:**
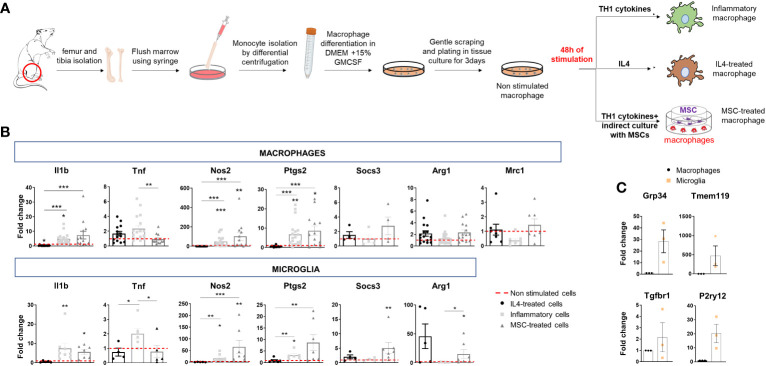
Polarization of bone marrow-isolated macrophages and expression of inflammatory and pro-regenerative markers. **(A)** Schematic representation of macrophage differentiation and polarization *in vitro*. **(B)** Gene expression of inflammatory markers (*Il1b, Tnf, Nos2 and Ptgs2*) and pro-regenerative markers (*Socs3, Arg1, Mrc1*) in macrophages (top) and microglia (bottom) under the same experimental conditions: IL4 treatment, stimulation with inflammatory cytokines (inflammatory cells) or inflammatory cytokines and MSCs (MSC-treated cells). Data are normalized to non-stimulated cells (fold change of 1) (macrophages, Kruskal–Wallis test with Dunn’s multiple comparison: *Il1b* p<0.0001 number of independent experiments N=13; *Tnf* p=0.0024 N=12; *Nos2* p<0.0001 N=12; *Ptgs2* p<0.0001 N=13; *Socs3* p=0.3221 N=4; *Arg1* p=0.0414 N=14; *Mrc1* p=0.0398 N=7. microglia, Kruskal–Wallis test with Dunn’s multiple comparison: *Il1b* p<0,0001 N=5; *Tnf* p=0.0071 N=4; *Nos2* p<0,0001 N=5; *Ptgs2* p=0.0005 N=6; *Socs3* p=0.0080 N=4; *Arg1* p=0.0026 N=5). **(C)** Low gene expression of homeostatic microglia markers (*Gpr34, Tmem119, Tgfbr1, P2yr12*) in bone marrow-derived macrophages compared to microglia in primary cultures (N=3/group; *Gpr34 t-test p=0.0493, Tmem119 t-test p=0.1397* and *P2ry12 t-test p=0.0419* p=0.1000, *Tgfbr1*, t-test p=0.4142).

Macrophages were established from the bone marrow of adult Sprague–Dawley rats (Charles River, Lecco, Italy) according to (20). After cutting off rat hind legs and removing skin, flesh and muscles, femur and tibia were washed with sterile PBS and cut at the joint. Using a syringe, bone marrow cavity of bones was flushed with DMEM medium (Life Technologies, Monza, Italy). Medium was collected, filtered through a 70µm Nylon cell strainers and centrifuged at 450 x g for 10 min at 4°C. Pellets were then dissociated in red blood cell lysis buffer (Lonza, Basel, Switzerland) for 30 min, diluted in cold DMEM medium and centrifuged at 450 x g for 10 min at 4°C. Resident macrophages were eliminated by adhesion to 100 nm-dishes for 4h at 37°C. Monocytes in the supernatants were centrifuged and cultured in 100 mm dishes in differentiating medium for 7 days (DMEM supplemented with 10% FBS (Life Technologies, Monza, Italy), glutamine (2mM; EuroClone, Milan, Italy) and 15% of medium (IMDM supplemented with 5% FBS, 2mM glutamine, 100U/ml penicillin and 100µg/ml Streptomycin) conditioned by X-63 cells for 4 days (as a source of GM-CSF). Macrophages were then gently scraped and re-plated on tissue-culture dishes in complete medium (DMEM supplemented with 10% FBS, 2mM glutamine, 100U/ml penicillin, 100µg/ml Streptomycin and 15% X-63 conditioned medium) (**Figure 1A**). One day after plating, macrophages were not stimulated (NS) or treated with Th1 cytokines dissolved in water and pre-diluted in medium before addition to cells to reach the final concentrations, i.e 40 ng/ml (IL-1β Peprotech, Milan, Italy), 40 ng/ml TNF (Peprotech, Milan, Italy) and 50 ng/ml IFN-ɣ (Sigma Aldrich, Milan, Italy), or with 40 ng/ml IL-4 (R&D, Milan, Italy) for 48h. Moreover, macrophages were indirectly cultivated with mesenchymal stem cells (MSCs) at a macrophages-to-MSCs ratio of 1:1 for 48h in macrophage medium supplemented with Th1 cytokines (**Figure 1A**). MSCs were plated on the insert and macrophages in the bottom chamber of the transwell (8 μm pore size filter; Constar, Corning, NY, USA). The insert was then removed, and macrophages used as a source of EVs.

#### 2.1.2 Microglia

We established mixed glial cell cultures, composed of astrocytes and microglia, from Sprague–Dawley rat pups (P2) and maintained the cells for 10 days in glial medium (MEM supplemented with 20% glucose, 100U/ml penicillin, 100µg/ml Streptomycin and 20% of South American fetal bovine serum (Life Technologies, Monza, Italy) to optimize microglia expansion. We harvested microglia by shaking mixed glial cultures for 40 min and re-plated the cells on poly-l-ornithine-coated culture dishes (50 μg/ml; Sigma-Aldrich, Milan, Italy). Microglia were kept in the absence of stimuli (NS) or stimulated with the same cocktail of Th1 cytokines used for macrophages.

#### 2.1.3 OPCs

We isolated OPCs from rat (Sprague-Dawley) cerebral cortex at postnatal day 2 (P2) by magnetic-activated cell sorting (MACS) (Miltenyi Biotec, Bergisch Gladbach, Germany) utilizing a Papain-based Neural Tissue Dissociation Kit (Miltenyi Biotec, Bologna, Italy) and anti-A2B5 magnetic microbeads (Miltenyi Biotec, Bologna, Italy). The cells were plated onto poly-d,l-ornithine-coated glass coverslips in Neurobasal (Life Technologies, Monza, Italy) supplemented with 2% B27 (Life Technologies, Monza, Italy), l-glutamine (2 mM; EuroClone, Milan, Italy), human platelet-derived growth factor (PDGF_BB_, 10 ng/ml; Sigma-Aldrich, Milan, Italy), and human basic fibroblast growth factor (bFGF, 10 ng/ml; Space Import Export, Milan, Italy), to promote proliferation (proliferating medium). 3 days after, we detached OPCs with accutase (Millipore, Burlington, MA, USA) for migration analysis or incubated the cells with a differentiating medium, i.e. Neurobasal medium devoid of growth factors and containing triiodothyronine T3 (10 ng/ml; Sigma Aldrich, Milan, Italy).

#### 2.1.4 Dorsal root ganglion -OPC co-cultures

We established DRG-OPC co-cultures as previously described (19). DRG were pulled out from spinal cord of E14.5 mouse embryos, put in culture (1 DRG/coverslip) in Neurobasal supplemented with 2% B27 and nerve growth factor (NGF) (100 ng/ml; Harlan, Milan, Italy) and cycled with fluorodeoxyuridine (10 μM; Sigma Aldrich, Milan, Italy) to remove non-neuronal cells. After three weeks, when neurites were well extended radially from DRG, we added 35 × 10^3^ OPCs to each DRG explant and maintained the explant in MEM (Life Technologies Monza, Italy) supplemented with glucose (4 g/l; Sigma Aldrich, Milan, Italy), 10% FBS and l-glutamine (2 mM; EuroClone, Milan, Italy). To induce myelination, recombinant chimeric tyrosine kinase receptor TrkA Fc (1 μg/ml; Sigma Aldrich, Milan, Italy) was added to the culture medium the following day.

#### 2.1.5 Mesenchymal stem cells

We prepared and expanded MSCs as described previously (21). We flushed out bone marrow cells from tibias and femurs of 6-8-week-old C57BL/6J mice and kept the cells in plastic culture dishes in Mesencult medium (Stem Cell Technologies, Vancouver, BC, Canada) until a confluency of 80%. The cells were then detached using trypsin and 0.02% EDTA (Euroclone, Milan, Italy) and plated in 75 cm^2^ flasks at the density of 4 × 10^5^ cells. After four to five passages in culture, mature MSCs were characterized by the expression of CD9, Sca-1, CD73, and CD44 and the lack of surface CD45, CD34, and CD11b. We verified their immunosuppressive activity in T-cell proliferation assays (21).

### 2.2 RNA isolation and qRT-PCR

Total RNA extracted from rat macrophages and microglia using Direct-zol™ RNA MiniPrep (Zymo Research, Irvine, CA, USA) was used for cDNA synthesis using High Capacity cDNA Reverse Transcription Kit (Applied Biosystems, Foster City, CA, USA) and Random Hexamers as primers. 80 ng of cDNA was amplified using TaqMan® Gene Expression Assay (Applied Biosystems, Foster City, CA, USA) and a QuantStudio™^5^ (ThermoFisher Scientific, Waltham, MA, USA) real-time PCR system. The mRNA expression was normalized to the reference gene Rpl13 (Ribosomal Protein L13) mRNA. Data obtained were quantified using the 2−ΔΔCT method. The list of primers used can be found in **Table 1**.

**Table 1:** List of gene expression assays for qPCR.

Gene symbol	Name	Taqman assay
*Il1-β*	Interleukin 1 Beta	Rn00580432_m1
*Tnf*	Tumour Necrosis Factor	Rn99999017_m1
*Nos2*	Nitric Oxide Synthase 2	Rn00561646_m1
*Ptgs2*	Cyclooxygenase 2	Rn01483828_m1
*Socs3*	Suppressor Of Cytokine Signaling 3	Rn00585674_s1
*Arg1*	Arginase 1	Rn00691090_m1
*Mrc1*	Mannose receptor 1	Rn01487342_m1
*Gpr34*	G Protein-Coupled Receptor 34	Rn02585733_s1
*Tmem119*	Transmembrane Protein 119	Rn01480631_m1
*Tgfbr1*	Transforming Growth Factor Beta Receptor 1	Rn00688966_m1
*P2ry12*	Purinergic Receptor P2Y12	Rn02133262_s1
*Rpl13a*	Ribosomal Protein L13	Rn00821946_g1

### 2.3 Calcium imaging

Intracellular calcium (Ca^2+^) levels were monitored in macrophages loaded with Fura-2/AM dye (Merck, Darmstadt, Germany) for 40 min at 37°C (22). Cells were then washed in Krebs–Ringer’s HEPES solution (KRH; 125 mM NaCl, 5 mM KCl, 1.2 mM MgSO4, 1.2 mM KH2PO, 2 mM CaCl2, 6 mM d-glucose, and 25 mM HEPES/NaOH, pH 7.4) and placed in the recording chamber of an inverted microscope (Axiovert 100, Zeiss, Oberkochen, Germany) equipped with a Ca^2+^ imaging unit. We used Polychrome V (TILL Photonics GmbH, Graefelfing, Germany) as light source for excitation at 340 and 380 nm wavelengths. We collected fluorescent images with a CCD Imago-QE camera (TILL Photonics GmbH, Gräfelfing, Germany) and analysed Ca^2+^ dynamics with FEI Live Acquisition 2.6.0.14 software. Ca^2+^ concentration was expressed as F340/380 fluorescence ratio. To obtain temporal analysis of Ca^2+^ concentration ratio values were calculated from sequences of images in selected region of interest (ROI).

### 2.4 EV isolation and quantification by NTA

EVs were obtained from both non-stimulated macrophages/microglia and cells treated with IL-4/inflammatory cytokines and/or MSCs. To isolate EVs, cells were thoroughly washed to remove damaged cells and then exposed to 1 mM ATP (Merck, Milan, Italy) in KRH for 30 min to enhance EV production. As described for microglia (23), short time macrophage conditioning under ATP stimulation in KRH minimizes collection of dying cells or broken cell membranes, while ensuring a good EVs yield. Conditioned KRH was harvested and centrifugated twice at 300 ×g for 10 min at 4°C to remove cells and debris. Total EV population (small and large EVs) was pelleted at 100000 ×g for 1h at 4°C and re-suspended in 800 μl of KRH filtered 0.1 μm. The concentration and dimension of EVs were evaluated using NanoSight NS300 (NanoSight, Salisbury UK) equipped with SCMOS camera and a 488 nm laser. Four recordings of 30 sec were performed for each sample and collected videos were analyzed using the NTA-software (version 2.3), with automatic set of minimal expected size, minimal track length and blue setting. Speed of camera shutter was set at 15 ms and camera gain was fixed to 300. Room temperature parameter was varying from 25°C to 28°C.

### 2.5 Lipid extraction from EVs

Total lipids were extracted using a 2:1 (by volume) of chloroform: methanol solution as previously described (24). To obtain multilamellar vesicles, lipid fraction was evaporated under a stream of nitrogen, dried for 1h at 50°C and dissolved in PBS at 40°C. Multilamellar vesicles were sonicated in order to obtained small unilamellar vesicles, following the procedure of (25).

### 2.6 Raman spectroscopy

The Raman analysis was performed as previously described (26). Briefly, freshly isolated EVs were air dried on a calcium fluoride slide and all measurements were performed with a Raman microspectroscope (LabRAM Aramis, Horiba Jobin–Yvon S.A.S, Lille, France) equipped with a 532 nm laser line and a Peltier-cooled CCD detector. Acquisitions were performed with 50X objective (NA 0.75, Olympus, Tokyo, Japan), 400 μm entrance slit, 1800 grooves mm^−1^ diffraction grating, and confocal mode (600 μm pinhole) in the spectral ranges 600–1800 cm^−1^ and 2600–3200 cm^−1^. Accumulation times were 2 × 30 sec per spectrum. Instrument calibration was performed using silicon reference sample. For every EV type and for lipid standards, at least 30 independent replicates were obtained. Polynomial baseline correction and vector normalization were performed after acquisition using Labspec6 (Horiba) and Origin2021 (OriginLab, Northampton, MA, USA) before the multivariate statistical analysis. For the analysis of lipids, standards were resuspended in NaCl 0.9%, and spectra were obtained with the same acquisition parameters as EV samples.

### 2.7 Isolation of myeloid EVs from human plasma

Venous blood (7–10 ml) was collected into 0.5 ml of saline with EDTA and centrifuged at 1500× g for 15 min at 4°C. Plasma aliquots were stored at −80°C pending use. Total EV isolation from plasma was performed with Total Exosome Isolation kit (Invitrogen, Waltham, MA, USA) according to the manufacturer’s instructions. Briefly, 500 μl of plasma were centrifugated at 10000xg for 20 min at RT to remove cellular debris, and total EVs were precipitated from 125 μl of supernatants using 37.5 µl of Exosome Precipitation Reagent (Invitrogen, Waltham, MA, USA). After 10 min incubation and 20 min centrifugation at 10000x g at RT, each total EV pellet was diluted in 100 μl of phosphate buffer. For myeloid EVs enrichment, total EVs suspensions were incubated for 4 h at 4°C with 4 μg of biotinylated Isolectin B4 (IB4; 1 mg/ml; Invitrogen, Waltham, MA, USA) diluted in 50 μl of 3% BSA, and then with 15 μl of streptavidin Ultralink resin (Thermo Scientific, Rockford, IL, USA) and 25 μl of 3% BSA. Pellets, re-suspended in 200 μl of 0.1 M glycine solution (pH 3.0), were centrifuged for 5 min at 4500×g at 4°C to separate myeloid IB4+ EVs from the bead-antibody complex (27). Supernatants containing IB4+ EVs were transferred to tubes containing 15 μl of 1 M TRIS-HCl (pH 8.0) and stored at − 80°C until immediately prior to assays.

Patients referred to the MS Center of IRCCS Fondazione Ca’ Granda Ospedale Maggiore Policlinico, Milan. Plasma was obtained from three patients affected by relapsing remitting MS (RRMS). They were two female and one male (mean age ± SD: 51 ± 17). Diagnosis was performed according to the 2010 McDonald criteria (28). Before the blood withdrawal, patients were not under steroid treatment for at least four weeks. The patients provided written informed consent to participate in research studies.

### 2.8 Sample preparation and (UHPLC)-mass spectroscopy/mass spectroscopy system

Samples were dried under flowing nitrogen gas, then eCBs were extracted by the addition of 0.8 ml of ethanol per tube. The tubes were then vortexed, for 5s, shaken at 1500 RPM for 5 min at 4°C, vortexed, for 5s, and subsequently centrifuged at 12,000 g for 5 min at 4°C. Next, the supernatants were evaporated to dryness using a speedvac cold trap concentrator. Each dried sample was reconstituted in a mixture of 30 μl MeOH/H2O (1:1, v/v) and transferred to glass vials with micro-inserts, capped immediately, and injected into the Ultra High-Performance Liquid Chromatography (UHPLC)- Mass Spectroscopy/Mass Spectroscopy system. From each sample, 5 μl were injected into our Liquid Chromatography - Mass Spectroscopy system.

Samples were analyzed in dynamic multiple reaction mode (dMRM) on an Agilent 1290 Infinity UHPLC system connected to an Agilent 6460 triple quadrupole (QqQ) mass spectrometer (MS Agilent Technologies Inc., Santa Clara, CA, USA). Waters HSS T3 2.1 × 100 mm, 1.8 μm column protected by a C18 HSS T3 VanGuard Pre-column (100Å, 1.8 µm, 2.1 mm × 5 mm) both from (Waters, Taastrup, DK.) used for separation of eCBs. The column temperature was maintained at 45°C throughout the run with a flow rate of 0.4 mL/min. Solvent A contained water and solvent B acetonitrile/isopropanol (67/33) (v/v). 0.1% formic acid (v/v) was added to both solvents. The following gradient was used for positive mode analysis: 0-1 min, 60% A, 1-2 min, ramping to 20% A, 2-8 min, 0% A, 8-9 min, 0% A, 9-9.2 min, ramping back to 60% A, 9.2-12 min, 60% A. Instrument-dependent parameters for mass spectrometry were as follows: The nitrogen drying gas temperature and flow was 325°C and 12 L/min, respectively. The capillary voltage was 3500. The nebulizer pressure was controlled at 45 psi. The nitrogen sheath gas flow and temperature were maintained at 11.0 L/min and 325°C, respectively. AEA and 2-AG quantification were performed based on an external calibration curve, using the following precursor ions: m/z 348.2 and m/z 379.2, and product ions (fragments) m/z 91.3 and m/z 105, respectively.

### 2.9 OPC migration assay

OPCs migration was performed as previously described (29). Briefly, Boyden chambers (8 μm pore size filter; Constar, Corning, NY, USA) was placed inside 24-well plates and OPCs (5 × 10^4^) in neurobasal medium (200 μl) were seeded into the upper well. The lower well was filled with the same medium (600 μl) containing EVs released from twice as many macrophages (1 × 10^5^ cells; ~ 6 × 10^8^ EVs/ml). The chemotactic factor sphingosine-1 phosphate (S1P) (100 nM) and EVs from inflamed microglia were used as positive controls. After 16 h, non-migrating cells were removed from the upper surface of the filter with a cotton swab, whereas the cells migrated to the lower side of the filter were processed by fixation in 4% paraformaldehyde followed by staining with Hoechst33258 (Life Technologies, Monza, Italy). Images were acquired at 20X magnification using an inverted fluorescence microscope (Zeiss, Oberkochen, Germany) and cells were counted in 45 random fields per well using ImageJ cell counter plugin. Data are expressed as a percentage of basal migration, i.e., the migration of OPCs without chemoattractant.

### 2.10 OPC differentiation assay

After culturing in proliferating medium for 3 days, OPCs were kept in differentiating medium for 24h and then exposed to EVs derived from twice as many macrophages for 48h (6 × 10^7^ EVs/ml). Cells were fixed and stained for anti-G Protein-Coupled Receptor 17 (GPR17) and anti-myelin basic protein (MBP) antibody (**Table 2**) in Goat Serum Dilution Buffer (GSDB; 20 mM sodium phosphate buffer, 450 mM NaCl, pH 7.4, 0.3% Triton X-100, 15% goat serum), followed by Alexa Fluor conjugated secondary antibodies (1:200; Molecular Probes, Life Technologies, Carlsbad, CA, USA). Differentiation towards mature oligodendrocytes was assessed by counting MBP^+^ cells over total number of cells (DAPI^+^) in 35–45 fields per coverslip (3 coverslips/condition) using ImageJ software. Immature oligodendrocytes, the most abundant cells after 3 days in differentiating medium, were revealed using GPR17 staining.

**Table 2:** List of primary antibodies used for immunofluorescence analysis.

Antibody	Host	Supplier	Dilution
Hoechst 33258		Life Technologies (Monza, Italy)	1:10000
Anti-CNPase	Mouse	Millipore (Burlington, MA, USA)	1:100
Anti-GPR17	Rabbit	Cayman Chemical (Michigan, USA)	1:100
Anti-GSTpi	Rabbit	MBL International Corporation (Sunnyvale, CA, USA)	1:500
Anti-MBP	Rat	Millipore (Burlington, MA, USA)	1:200
4′,6-Diamidino-2-phenylindole DAPI		Molecular Probes (Life Technologies, Monza, Italy)	1:20000
anti- Neurofilaments (SMI31)	Mouse	Cell Signaling (Beverly, MA, USA)	1:500
anti- Neurofilaments (SMI32)	Mouse	Cell Signaling (Beverly, MA, USA)	1:500
Anti-Isolectin IB4, Alexa Fluor 488 conjugate		Life Technologies (Monza, Italy)	1:100
Anti-Iba1	Rabbit	Wako Chemicals (Neuss, Germany)	1:100

### 2.11 Myelin deposition assay

The assay was performed as in Lombardi et al, 2019 (14). Co-cultures of OPC-DRG were maintained in 1 μg/ml TrkA-Fc for 5 days, then exposed to EVs for 6-7 days (three additions of fresh EVs - 6 × 10^7^ EV/ml - on alternate days). OPCs were paraformaldehyde fixed at 12 days *in vitro* (DIV 12) after plating on DRG and stained for anti-high-molecular-weight neurofilaments (NF, SMI31 and SMI32 in **Table 2**) and anti-MBP antibodies in GSDB, and then detected by Alexa Fluor 488 or Alexa Fluor 555 secondary antibodies (1:600; Molecular Probes, Life Technologies, Carlsbad, CA, USA). DAPI was used to label nuclei. Dako mounting medium (Dako, Milan, Italy) was employed. Z-stack images of MBP- and SMI31-SMI32-positive cells were acquired at 40× magnification (6 fields/coverslip) on a confocal microscope, using ZEISS LSM Image Browser to obtain the myelination index by automate quantification. At each z level, images in the stack were merged and red-green double-positive pixels reaching a predefined intensity threshold were shown in white. The myelination index, indicative of the amount of myelin per axon, was calculated as the ratio between the MBP^+^/SMI^+^ co-localizing pixels and the total SMI^+^ pixel areas.

### 2.12 Flow cytometry

The whole procedure was performed on ice, using 4°C refrigerated (and pre-chilled) centrifuges and ice-cold solutions. For detection of OPC maturation markers, OPCs were permeabilized using the BD Cytofix/Cytoperm™ (BD Bioscience, Themo Fisher Scientific, Waltham, MA, USA) and stained for the intracellular markers with anti-glutathione S-transferase-pi (GST-Pi) and anti-2’,3’-Cyclic-nucleotide 3’-phosphodiesterase (CNPase) antibodies (**Table 2**). Alexa Fluor 647 or Alexa Fluor 488 (1:200; Molecular Probes, Life Technologies, Carlsbad, CA, USA) were used as secondary antibodies and live cells were discriminated with LIVE/DEAD Fixable Dead Cell Stains (Themo Fisher Scientific, Waltham, MA, USA). FACS Canto II machine (BD Biosciences) and FlowJo analysis software (TreeStar), were employed for FACS analysis.

### 2.13 Statistical analysis

For data analysis and graphic presentation Graph-Pad Prism software (GraphPad Software Inc., San Diego, CA, USA) was employed. Data are mean ± SEM from the indicated number of independent experiments (N) (independent OPC cultures or EV preparations) if not otherwise specified. All data sets were tested for normal distribution, and the appropriate statistical test chosen accordingly. Specific p values were indicated in figure legend. Differences were considered significant when p<0.05 and indicated by asterixis: * = p<0.05; ** = p<0.01; *** = p<0.001 and **** = p≤0.0001. Not significant differences are indicated by ‘ns’. Relatively to Raman spectra, Principal Component Analysis and Linear Discriminant Analysis (PCA-LDA) were performed by Origin2021 software. Leave-one-out cross-validation was employed to test the sensitivity, specificity, and accuracy of the classification model to distinguish EV phenotypes by the overall biochemical composition. The Classical Least Square (CLS) fitting analysis was performed using Labspec6.

## 3 Results

### 3.1 Transcriptional states of macrophages versus microglia *in-vitro*

We first analyzed the transcriptional states of macrophages, obtained from the bone marrow of adult rats (20), and compared them to primary microglia. Rat Macrophages (r-macrophages) were cultured in control conditions (non-stimulated), in the presence of inflammatory cytokines (Th1 cytokine cocktail) or a pro-regenerative cytokine (IL-4) for 48h. In addition, r-macrophages were undirectedly co-cultured with mouse mesenchymal stem cells (m-MSCs) in transwells with inflammatory cytokines (**Figure 1A**), a protocol that drives pro-regenerative functions in rat microglia (14). Transcriptomic analysis showed the acquisition of distinct phenotypes in the four conditions (**Figure 1B**). Despite downregulation of pro-inflammatory *Tnf* and a trend to upregulation of the protective markers *Socs3*, *Arg1* and *Mrc1* in m-MSC-treated r-macrophages compared to inflammatory cells, m-MSC-treated cells maintained pro-inflammatory traits (elevated *Il1b*, *Nos2*, *Ptgs2*), as observed in r-microglia exposed to the same stimuli [**Figure 1B** (14)]. Overall, the response of macrophages to the different stimuli was similar to that of microglia, while expression levels of homeostatic microglial markers were extremely low in macrophages (**Figure 1C**), indicating that microglia and macrophages maintain a distinct transcriptional profile in the *in-vitro* environment.

### 3.2 ATP-induced EV production from macrophages versus microglia

**Figure 2:**
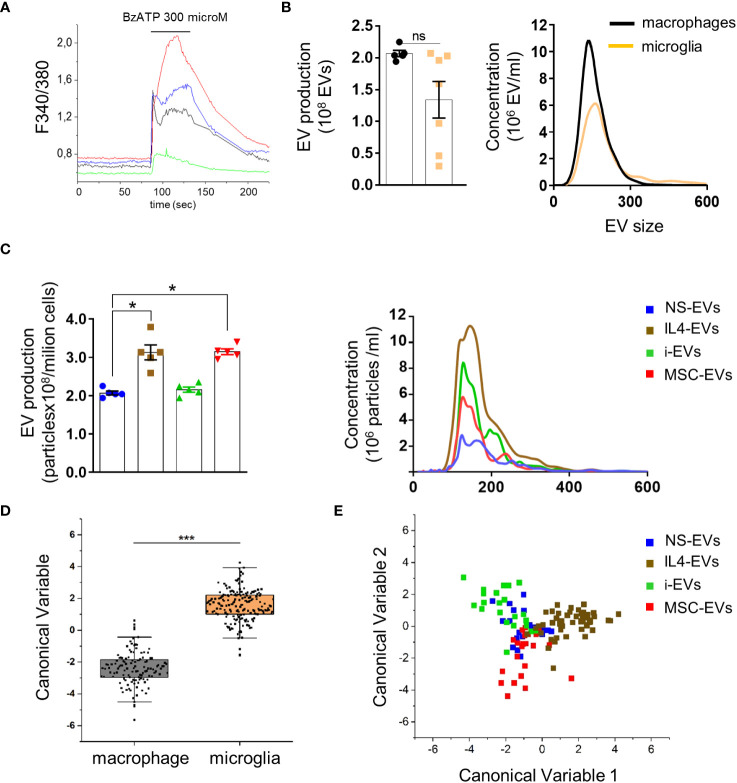
Quantification and Raman spectroscopy analysis of EVs produced by macrophages with different states versus microglia-derived EVs. **(A)** Temporal analysis of changes of intracellular calcium concentration, expressed as F340/380, in fura-2-loaded macrophages exposed to 300 μM BzATP (N of analyzed cells=50). Representative traces from four distinct macrophages are shown in distinct colors. **(B)** EV production (left) and size profile (right) from 1 x 106 non stimulated macrophages or microglia, quantified by NTA (N = 5 macrophages, N=7 microglia; Mann Whitney t-test p=0.0732). EV size, Mean±SEM: 176.0 ± 32.53 nm macrophages, 204.0 ± 50.91 nm microglia. **(C)** The graphs show production (left) and size distribution (right) of EVs from non-stimulated or polarised macrophages upon ATP stimulation (N=5; Kruskal-Wallis test p=0.0021 with Dunn’s multiple comparisons test). EV size Mean ± SEM: 185.3 ± 17.34 nm NS-EVs, 187.9 ± 32.88 nm IL4-EVs, 186.8 ± 23.90 nm i-EVs, 199.6 ± 40.09 nm MSC-EVs. **(D)** Canonical Variable scores obtained after PCA-LDA multivariate analysis of the Raman spectra obtained from macrophages and microglia. In the classification model, spectra from EVs were grouped based on the cell of origin to test Raman ability to discriminate the molecular composition of EVs from the two cell sources. The first 10 PC scores calculated by means of PCA were used for the LDA. Each dot represents a single spectrum (***p<0.001 after one way ANOVA). **(E)** Multivariate statistical analysis (PCA-LDA) performed on the Raman spectra of NS-EVs, IL-4-EVs, i-EVs or MSC-EVs derived from macrophages. The scatter plot represents the values obtained for the Canonical Variable 1 and Canonical Variable 2 after LDA. Each dot represents a single spectrum.

In immune cells expressing P2X7 receptor, ATP strongly enhances shedding of EVs from the cell surface (30–32). To assess the presence of functional P2X7 receptors macrophages were loaded with the ratiometric calcium dye Fura-2 and imaged upon persistent stimulation with 300 µM benzoyl-ATP (BzATP), a potent P2X7 receptor agonist (33). In virtually all cells (>98%), BzATP induced calcium rises, often made by a calcium peak followed by a calcium plateau (**Figure 2A**), reminiscent of those detectable in P2X7-expressing microglia and cortical astrocytes (34, 35), suggesting the expression of functional P2X7 receptors.

We next compared EV production between r-macrophages and r-microglia. Non-stimulated cells (both macrophages and microglia) were challenged with ATP for 30 min to enhance EV production. The supernatants were collected, pre-cleared from cellular debris and EVs were extracted by ultracentrifugation at 100,000 x g and quantified by Nanosight Tracking Analysis (NTA). No significant differences were found in EV size and production between macrophages and microglia (**Figure 2B**). Among macrophages-derived EVs, EVs from IL4-treated and m-MSC-treated cells (IL4-EVs, MSC-EVs) were higher in number compared to EVs from non-stimulated cells (NS-EVs) or inflammatory macrophages (i-EVs) (**Figure 2C**).

Next, we used Raman spectroscopy to get insight into the chemical composition of EVs released from macrophages in different states and compared them to microglial EVs. Raman spectroscopy provides information about the general chemical features of EVs in a single spectrum that accounts for all the biomolecular components present inside and outside EVs. Multivariate statistical analysis and linear discriminant analysis (LDA) revealed significant changes in the spectra of EVs from non-stimulated macrophages and microglia (**Figure 2D**). Raman spectroscopy could distinguish EVs produced by r-macrophages (any type) from those derived from r-microglia (any type) with an overall accuracy of 98.1% after cross-validation (p<0.001 after ANOVA). This indicates that the molecular composition of macrophage-derived EVs largely differs from that of microglial-EVs, despite the two cell types sharing a common myeloid origin and displaying a similar response to stimuli. Moreover, spectra of EVs from macrophages in response to different stimuli were distinguished from one another with good overall accuracy (88.2%, **Figure 2E**), although NS-EVs could be misclassified with i-EVs, IL-4-EVs or m-MSCs-EVs. This error in the group attribution can be visualized in **Figure 2E**, where NS-EVs dots overlap those of the other subclasses, suggesting the presence of shared components between the EVs released from untreated or stimulated macrophages. However, in the proposed classification model, IL4-EVs can be distinguished from NS-EVs, i-EVs and MSC-EVs with the lowest error rate (7.27% after leave-one-out cross-validation), suggesting peculiar features compared to the other subgroups.

### 3.3 Effects of macrophage-derived EVs on migration and differentiation of cultured OPCs

Our previous data indicated that EVs produced by r-microglia promote migration and differentiation of cultured rat OPCs (r-OPCs), independently of the transcriptional state of donor microglia (14).

**Figure 3:**
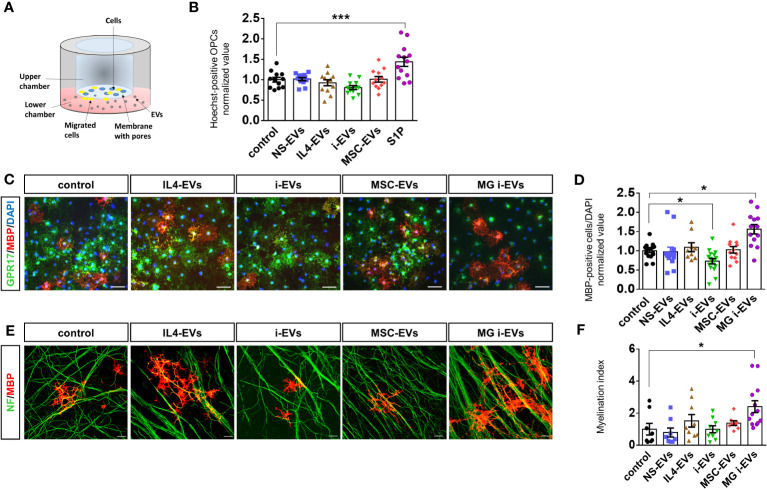
EVs derived from macrophages do not promote OPC migration and differentiation. **(A)** Scheme of the chemotactic assay. **(B)** The bar graph shows the percentage of migrated OPCs after an overnight exposure to NS-EVs, i-EVs, IL-4-EVs, or MSC-EVs from macrophages with respect to control OPCs. S1P was used as positive control. The number of migrated Hoechst+ cells was counted in 60 optical fields at 20X magnification (N=3, four replicates; One-way Anova p<0,0001 with Holm Sidak’s multiple comparisons test). **(C)** Representative images of MBP (red), GPR17 (green and DAPI (blue) staining in control OPCs or OPCs exposed to EVs derived from macrophages treated as in **(B)** (Scale bars, 50 μm). **(D)** Bar graphs shows quantification of MBP+ cells in control OPCs or OPCs exposed to EVs from macrophages with the four distinct phenotypes (N=4, three-fiver replicates; Kruskal-Wallis test p<0,0001 with Dunn’s multiple comparisons test). Microglia-derived i-EVs (MG i-EVs) were used as positive control. **(E)** Representative images of OPC-DRG co-cultures maintained in control conditions or exposed to IL4-EVs, i-EVs or MSC-EVs and stained for MBP (red) and neurofilament (NF, green) (Scale bars, 20 μm). **(F)** Myelination index (MBP staining/NF staining) under experimental conditions as in **(E)** (N=3, three replicates; Kruskal-Wallis test p=0.0069 with Dunn’s multiple comparisons test).

To explore how r-macrophage-derived EVs influence r-OPC migration, we exposed primary OPCs overnight to macrophage-derived EVs in a transwell-based migration chamber (**Figure 3A**). As positive control we used sphingosine 1 phosphate (S1P), a well-known chemoattractant (36) (**Figure 3B**). We found that macrophage-derived EVs did not alter OPC transit through the transwell filter, regardless of donor cell phenotypes (**Figure 3B**).

Next, we explored the effects of macrophage-derived EVs on OPC maturation. Postnatal r-OPCs were cultured for three days in proliferating medium, followed by one day in differentiating medium, and exposed to EVs from r-macrophages for two days, according to our previous study (14). OPCs were then fixed and stained for MBP, a myelin protein expressed in mature OLs, and for GPR17, a marker of immature OLs which is downregulated in mature cells. Nuclei were labelled by DAPI (**Figure 3C**). OPC differentiation was determined by analysis of MBP-positive over total DAPI-positive cells. EVs from inflammatory microglia (MG i-EVs) were used as positive control. The analysis revealed that EVs released by inflammatory macrophages (i-EVs) decreased the percentage of MBP-positive mature OLs compared to control cells and cells exposed to EVs from IL4-treated macrophages (IL4-EVs) (**Figures 3C, D**). No changes in the percentage of fully differentiated OLs were found in OPCs exposed to EVs from inflammatory macrophages co-cultured with MSCs (MSC-EVs) or non-stimulated macrophages (NS-EVs) compared to control (**Figures 3C, D**).

To corroborate these findings, OPC differentiation was quantified by flow cytometry analysis using CNPase, a marker of pre-OLs, and Glutathione S-transferase (GST-pi), a marker for mature OLs. The analysis confirmed that i-EVs inhibit OPC transition to CNPase-positive pre-OLs (CNPase median fluorescence intensity (MFI): 5950 control OPCs, 3598 i-EVs treated-OPCs; **Supplementary Figure 1A**) and GST-pi positive mature OLs (GST-pi MFI: 1490 control OPCs; 1154 i-EVs treated-OPCs; **Supplementary Figure 1**). In addition, it revealed that IL4-EVs may favor OPC transition to a more differentiated CNPase^+^ pre-oligo stage (CNPase MFI: 11015 IL4-EVs treated-OPCs; **Supplementary Figure 1A**), although not being able to promote differentiation to fully mature GST-pi^+^ myelin-forming OLs (GST-pi MFI: 1574 IL4-EVs treated OPCs; **Supplementary Figure 1B**).

We explored the action of EVs on myelin deposition in r-OPCs co-cultured with murine dorsal root ganglia (m-DRG) neurons, a convenient *in vitro* approach to study myelination (14, 37). We exposed r-OPCs to EVs derived from r-macrophages with different activation states for 6-7 days (three additions of EVs, on alternate days), fixed and immunostained the cells for MBP and neurofilaments to reveal axons and myelin respectively. Quantification of MBP^+^ segments extending along neurofilament^+^ axons (myelination index) did not show significant alteration of myelin deposition upon exposure to EVs (**Figure 3E, F**), despite a tendency towards increased myelin formation in cultures exposed to IL4-EVs.

Collectively, these data indicate that macrophage-derived i-EVs limit OPC maturation while IL4-EVs or MSC-EVs fail to promote transition to fully differentiated cells and to induce myelin deposition *in-vitro*. Of note, i-EVs derived from r-macrophages have an opposite action on r-OPC differentiation compared to i-EVs released by r-microglia, which enhanced r-OPC maturation (**Figure 3D**) (14). This finding suggests that macrophage-derived EVs could be used as reference for the identification of promyelinating components in microglial EVs by differential analysis of their lipid cargo.

### 3.4 Lipidic content characterization in macrophage- versus microglia-derived EVs by Raman spectroscopy

**Figure 4:**
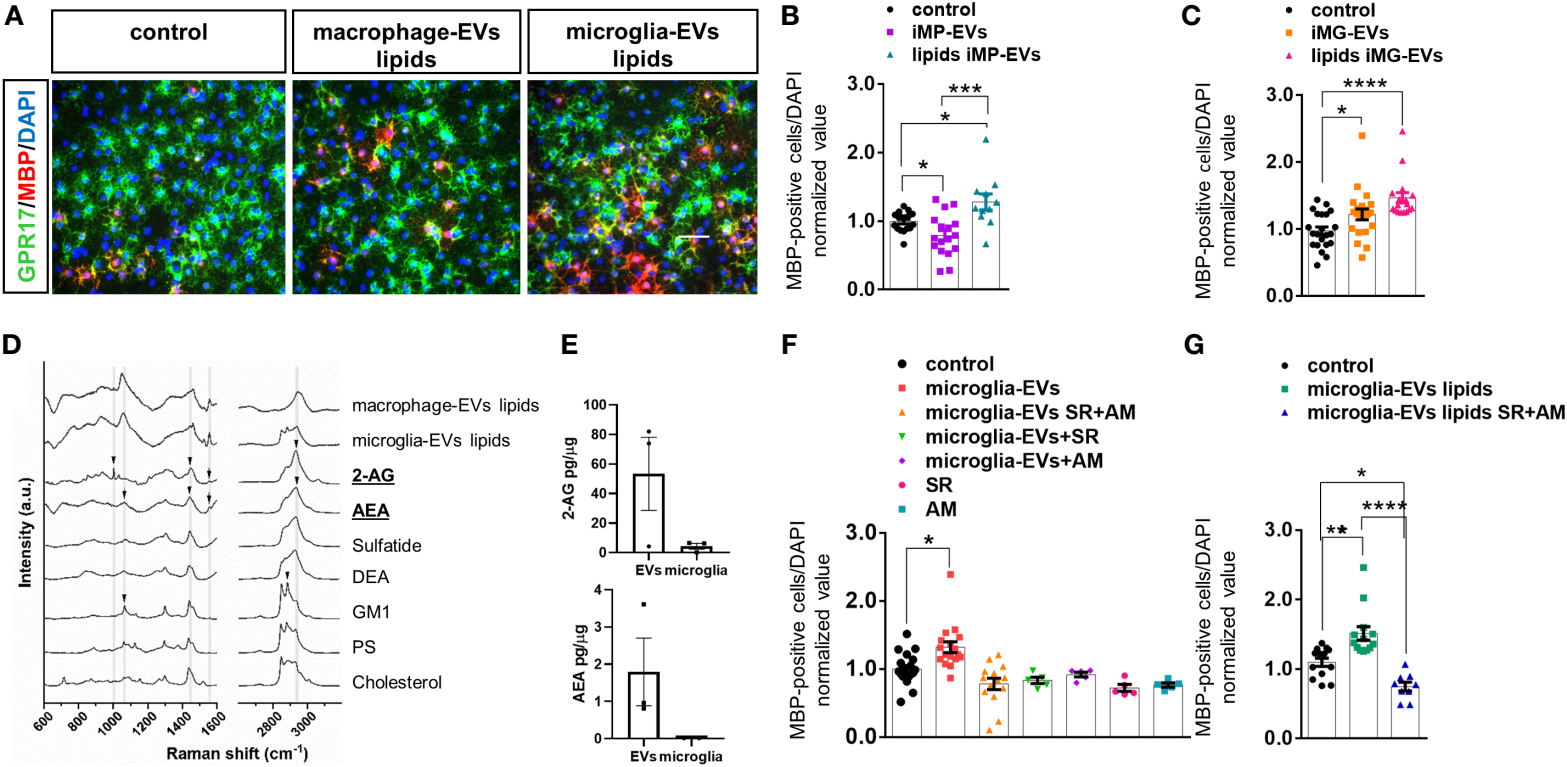
eCBs are present among EV lipids and drive OL differentiation. **(A)** Representative images of OPCs stained for MBP (red), GPR17 (green) and DAPI (blue) in non-stimulated cultures (control) or cultures exposed to the lipid fraction from EVs released by inflammatory macrophages (macrophage-EVs lipids) or microglia (microglia-EVs lipids). **(B, C)** Bar graphs shows quantification of MBP^+^ cells in control OPCs and in OPCs exposed to intact macrophage EVs (macrophage-EVs) or their lipid extract (macrophage-EVs lipids) (N=4 in two-three replicates; One-way ANOVA p=0,0002 with Holm Sidak’s multiple comparisons) **(B)** or to intact microglial EVs (microglia-EVs) or their lipid extract (microglia-EVs lipids) (N=5, three-four replicates; Kruskal-Wallis test p<0,0001 with Dunn’s multiple comparisons test) **(C)**. **(D)** Raman spectra obtained from lipid extracts of EVs derived from inflammatory macrophages (macrophage-EVs lipids) or microglia (microglia-EVs lipids) in comparison with the spectra of lipid standard (2-AG, AEA, Sulfatide, DEA, GM1, PS and Cholesterol). Arrows show the presence of peaks common to 2-AG, AEA and GM1 in the lipid extracts. **(E)** 2-AG and AEA content in EVs versus donor microglia (N=3). Values are normalized on sample protein content. **(F, G)** Bar graphs shows quantification of MBP^+^ cells in control OPCs, OPCs exposed to intact microglial EVs (microglia-EVs) or their lipid extract (microglia-EVs lipids) in absence or in presence of eCB receptor antagonists SR141716A and AM630 in combination (SR+AM) or alone (SR, AM) [**(F)** N=3, three replicates; One-way ANOVA p<0,0001 with Holm Sidak’s multiple comparisons; **(G)** N=3, three replicates; One-way ANOVA p<0,0001 with Tukey’s multiple comparisons]. * = p<0.05; ** = p<0.01; *** = p<0.001 and **** = p≤0.0001. Not significant differences are indicated by ‘ns’.

Given that the promyelinating action of microglia-derived EVs was previously attributed to their lipid components (14), we analyzed the impact of lipids extracted from macrophage-derived i-EVs on OPCs. Unexpectedly, we found that unlike intact i-EVs, which blocked OPC maturation, the lipid component of macrophage-derived i-EVs significantly enhanced OPC differentiation (**Figures 4A, B**) in a similar manner to microglia-derived EVs (**Figures 4A–C**). This suggested that macrophage-derived i-EVs may share with microglial EVs some lipid component(s) with pro-differentiating activity.

Next, we took advantage of Raman spectroscopy to search for common promyelinating lipid(s) in i-EVs produced by either microglia or macrophages. We employed a candidate approach and looked for endogenous cannabinoids (eCBs), i.e. 2-arachidonoylglycerol (2-AG) and anandamide (AEA), in light of their well-known capacity to promote OPC differentiation (17, 38, 39). Activation of either cannabinoid receptor type 1 (CB1R) or cannabinoid receptor type 2 (CB2R) by eCBs has been shown to increase OPC branching and MBP accumulation (17, 40, 41), while pharmacological inhibition of cannabinoid receptors prevented OPC maturation (41–43).

The comparison of Raman spectra obtained from lipid extracts of i-EVs derived from macrophages and microglia with lipid standards (2-AG, AEA, sulfatide, docosatetraenoyl ethanolamide (DEA), Ganglioside (GM1), phosphatidylserine (PS) and cholesterol) demonstrated that both cell types release EVs that contain eCBs. Specifically, the comparison of spectra highlighted the presence of peaks common to 2-AG and AEA in the lipid extracts (**Figure 4D**, **Supplementary Figure 2**). Besides, CLS fitting was performed on the spectra from lipid extracts of i-EVs derived from both macrophages and microglia, demonstrating the contribution of AEA to the spectra of both samples, as well as 2-AG, sulfatide, and DEA, which appear to be present, albeit to a different extent, in the two cell sources (data not shown).

The presence of 2-AG and AEA in EVs from unstimulated microglia was further indicated by target Ultra High-Performance Liquid Chromatography (UHPLC)-MS/MS quantification. This analysis revealed a 2-AG content of approximately 61.97± 28.66 picograms and about 2.08± 1.06 picograms of AEA in EVs derived from 1 million microglia with an enrichment of about 12 folds for 2-AG and 200 folds for AEA in EVs compared to donor cells once normalized on protein amount (**Figure 4E**).

### 3.5 2-AG and AEA content in human plasma myeloid EVs

To assess the eCBs content in EVs produced by microglia/macrophages *in vivo*, we finally quantified 2-AG and AEA in myeloid EVs circulating in the plasma of a pool of three patients (1.25 ml plasma in total) with relapsing/remitting MS. Total plasma EVs were first isolated by a commercial kit (Total Exosome Isolation kit, Invitrogen) and then microglia/macrophage-derived EVs were extracted by immune-affinity capture using IB4, a myeloid marker (27). In IB4+EVs derived from 1 ml of plasma, we found detectable levels of both 2-AG (37.2 picograms) and AEA (3.6 picograms), accounting for about 26% and 54% of 2-AG and AEA content in total plasma EVs, respectively.

### 3.6 The eCB cargo of EVs promotes oligodendrocyte maturation

To prove the involvement of eCBs released in association with EVs in OPC maturation, we next exposed OPCs to either intact i-EVs from microglia or their lipid extracts in the presence of the eCB receptor antagonists SR141716A (1µM) and AM630 (1µM), that block CB1R and CB2R, respectively, or vehicle. Analysis of MBP-positive OLs revealed that full blockade of eCB signalling, achieved by combined exposure to SR141716A and AM630, completely abolished the pro-differentiating activity of intact microglial i-EVs (**Figure 4F**) and their extracted lipids (**Figure 4F**), and decreased OPC differentiation induced by EV lipids below control condition (**Figure 4G**). Notably, also the single exposure to either SR141716A or AM630 was sufficient to prevent EVs-induced OPC differentiation and tended to decrease spontaneous OPC differentiation in the absence of EVs (**Figure 4F**).Taken together these data indicate that both AEA and 2-AG are released by microglia and macrophages via EVs and promote OPC differentiation likely interacting with CBR. In addition, they confirm previous evidence indicating an autocrine/paracrine activation of CB1R and CB2R by eCBs endogenously released by OPCs (44) that critically supports OPC differentiation to myelin forming cells.

## 4 Discussion

### 4.1 Inflammatory macrophages exert detrimental effects on OPCs via EVs

In the present study, using a simple *in-vitro* approach, we show that macrophages differ from microglia in the way they act on OPCs through EV release. Macrophage-derived EVs do not promote OPC migration and differentiation, or *in-vitro* myelin deposition by OLs, even in response to IL-4 or co-culturing with MSCs (i.e. pro-regenerative stimuli). Conversely, microglia-derived EVs favor OPC differentiation and myelin formation when added to cultured OPCs [this study and (14)]. These findings suggest that infiltrating macrophages may have a direct detrimental action on OPCs at myelin lesions through their released EVs, whereas microglia-derived EVs favor OPC migration and differentiation, promoting myelin repair [this study and (14, 45, 46)]. Despite being obtained *in-vitro*, using polarizing stimuli that cannot fully mimic the dynamic and multidimensional states of microglia in the white matter, these data may help to distinguish the differential contribution of infiltrating macrophages versus brain-resident microglia to myelin lesion and repair, a topic that is still largely unclear.

Advancements in single-cell RNA seq technologies and genetic fate mapping have ameliorated our ability to distinguish macrophages from microglia at focal myelin lesions in MS (47–49). However, the high plasticity of myeloid cells, which change their signatures in the course of the disease (50, 51), makes it still challenging to discriminate between the two cells and their distinct functions at myelin lesion sites (47, 49). An additional level of complexity comes from recent studies showing that microglia and macrophages may differentially modulate each other’s functions during demyelination and remyelination. Indeed, after lysophsosphatidylcholine-induced lesions, microglia, which dominate the lesion site, were reported to limit infiltration of macrophages (48), while infiltrating macrophages modulate microglia-mediated phagocytosis and inflammation that drive brain damage (52) and their functional state influences microglial responses (53).

In our study, microglia and macrophages maintain a distinct transcriptional profile in the *in-vitro* environment. However, analyses in a more physiological settings are necessary to validate the distinct action of macrophages- and microglia-derived EVs on the remyelination process.

### 4.2 The lipid cargo of inflammatory macrophage-derived EVs promotes OPC differentiation

A key finding of this study is the demonstration that the lipid component of macrophage-derived EVs promotes OPC maturation similarly to that of microglial EVs. Indeed, biochemical fractionation experiments and immunocytochemical analysis of cultured OPCs show that the lipid extract of EVs released by cytokines-treated macrophages increases the percentage of MBP^+^ myelin forming OLs similarly to microglial EVs and their lipids, while intact macrophage-derived EVs significantly inhibit OPC maturation. This unexpected result did not allow us to use macrophages-derived EVs as a negative reference sample for the identification of promyelinating lipids in microglial EVs and suggests that other components of inflammatory macrophage-derived EVs, such as the protein and/or nucleic acid cargo, may inhibit maturation of cultured OPCs, overcoming the promyelinating action of EV lipids.

Consistent with functional data, comparative analysis of the chemical content of EVs by Raman spectroscopy (26) shows major differences between EVs derived from macrophages or microglia, while highlighting similarities (shared peaks that mirror common molecules) in the lipid extracts from the two EV types. Specifically, the endocannabinoids AEA and 2-AG, along with sulfatide and DEA, were detected in both EV populations. Additional quantification of endocannabinoids by LC-Mass Spectrometry shows a strong enrichment in AEA and 2-AG in microglial EVs compared to donor cells and validates the presence of both endocannabinoids in myeloid EVs isolated from human plasma.

### 4.3 AEA and 2-AG in microglial EVs promote OPC differentiation

AEA and 2-AG are endogenous signaling lipids previously involved in protection of neuronal integrity (54, 55), modulation of immune cell-mediated inflammatory response (56–58) and myelin preservation (43, 59) in multiple sclerosis (MS) and models of experimentally induced demyelination.

Neuroprotective effects of eCBs, such as inhibition of neuron hyperactivity, have been ascribed to CB1R activation, while immunomodulatory effects have been linked to CB2R stimulation (17). However, the relative contribution of CB1R and CB2R in preventing pathogenic events in MS is not completely defined. This holds true especially for degenerative processes affecting OLs, where CB1R and CB2R coexist (40), change their expression during development (17), and are activated by 2-AG, AEA and other endocannabinods (with different affinities).

In this study, usingCB1R and CB2R antagonists, we prove the involvement of endocannabinoid signaling in the promyelinating action of microglia-derived EVs and identify endocannabinoids as important mediators in microglia-oligodendrocyte crosstalk, favoring myelin formation. Not only do the combination of the two antagonists nullify the pro-differentiating activity of microglial EV lipids, but they also decrease OPC maturation below control values, as evidenced by lower percentage of MBP-positive OLs. The latter finding is consistent with previous works showing that OLs constitutively release 2-AG, which promotes OPC proliferation and maturation via autocrine/paracrine activation of CB1R and CB2R, and blockade of CB1R and CB2R decreases OPC maturation *in-vitro* (16) and exacerbate myelin loss in demyelination mouse models (59–62). Importantly, our study suggests that exacerbation of myelin lesions in mice treated with CB1R and CB2R antagonists may result from both inactivation of the eCB tone governed by synthetic/degradative pathways in OLs and eCBs released by microglia via EVs.

The pro-myelinating action of microglial EVs appears to be mediated by both eCB receptors, given that both CB1R and CB2R antagonist prevented EVs-induced OPC differentiation and their combination significantly inhibited OPC maturation below control levels. Both eCBs are present in the lipid extracts of microglia/macrophage-derived EVs and discerning their specific effects is difficult due to common receptor signaling (similar affinity of 2-AG for CB1 and CB2; higher affinity of AEA for CB1).

The identification of eCBs as active lipid components of microglial EVs indicates that pro-myelinating lipids released by microglia act as signaling molecules that promote myelin formation. However, we cannot exclude that other lipid components of microglial EVs may contribute to OL maturation acting as substrates (building blocks) for myelin synthesis.

## 5 Conclusions

The present study, by showing that eCBs released by microglia and macrophages via EVs promote OPC differentiation, highlights the role of these bioactive lipids in myeloid cells-OLs signaling, and rekindles interest in endocannabinoids and their receptors (CB1R/CB2R/other receptors) for new therapies fostering myelin repair (41, 63).

## Data availability statement

The raw data supporting the conclusions of this article will be made available by the authors, without undue reservation.

## Ethics statement

The studies involving humans were approved by Institutional Review Board (IRB) of the Fondazione IRCCS Ca’ Granda Ospedale Maggiore Policlinico (Milan, Italy). The studies were conducted in accordance with the local legislation and institutional requirements. The human samples used in this study were acquired from primarily isolated as part of your previous study for which ethical approval was obtained. Written informed consent for participation was not required from the participants or the participants’ legal guardians/next of kin in accordance with the national legislation and institutional requirements.The animal study was approved by Italian Ministry of Health, Authorization: 1112-2016PR. The study was conducted in accordance with the local legislation and institutional requirements. The patients provided written informed consent to participate in this study.

## Author contributions

ML: Data curation, Formal analysis, Investigation, Writing – review & editing, Visualization. FS: Data curation, Formal analysis, Investigation, Writing – review & editing. MG: Data curation, Formal analysis, Funding acquisition, Investigation, Writing – review & editing, Visualization. SR: Data curation, Formal analysis, Investigation, Writing – review & editing, Visualization. EB: Data curation, Formal analysis, Investigation, Writing – review & editing. FF: Data curation, Formal analysis, Investigation, Writing – review & editing. PG: Data curation, Formal analysis, Investigation, Writing – review & editing. SP: Data curation, Formal analysis, Investigation, Writing – review & editing. NR: Writing – review & editing, Conceptualization, Supervision. AU: Writing – review & editing, Conceptualization, Supervision. MG: Data curation, Formal analysis, Writing – review & editing. GD: Data curation, Formal analysis, Writing – review & editing, Investigation. TR: Writing – review & editing, Conceptualization, Supervision. KH: Data curation, Formal analysis, Investigation, Writing – review & editing. CL-Q: Writing – review & editing, Conceptualization, Supervision. CF: Writing – review & editing, Conceptualization, Supervision. AG: Data curation, Investigation, Methodology, Writing – review & editing. MF: Funding acquisition, Writing – review & editing, Conceptualization, Supervision. CV: Conceptualization, Funding acquisition, Supervision, Writing – original draft, Writing – review & editing.
