# Supplementary material for: Extracellular vesicles released by microglia and macrophages carry endocannabinoids which foster oligodendrocyte differentiation
Source: Front Immunol. 2024 Feb 23;15:1331210. doi: 10.3389/fimmu.2024.1331210 (PMC10921360; doi:10.3389/fimmu.2024.1331210)
Supplement: Supplementary file 1 [file DataSheet_1.pdf]

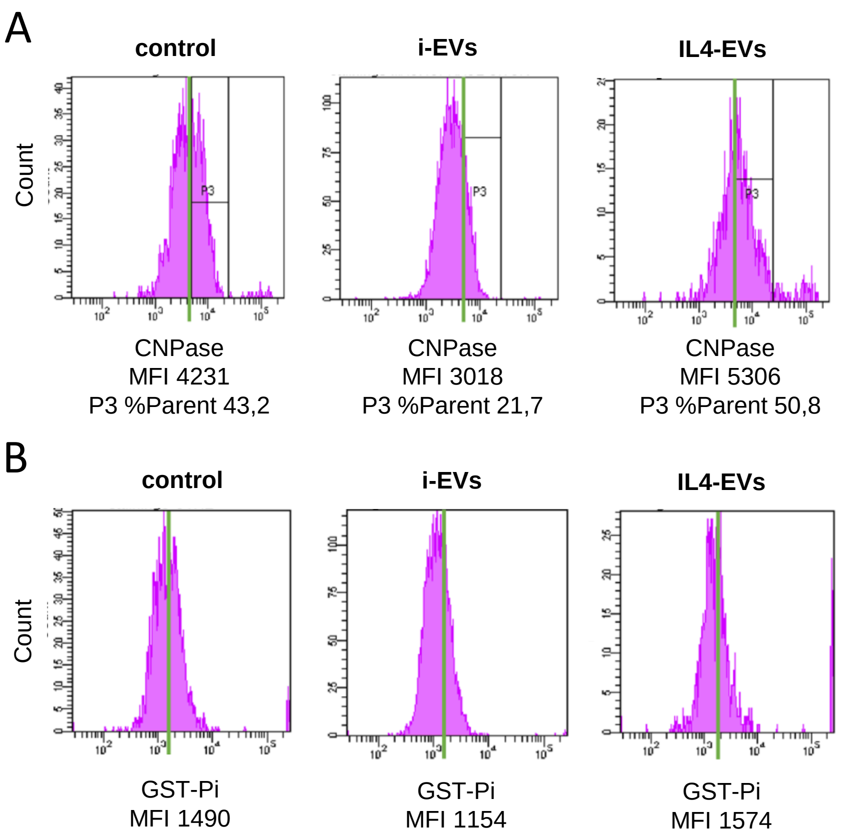

Suppl. Fig 1 EVs derived from macrophages fail to promote complete OPC differentiation. (A) Histograms showing the mean fluorescence intensity (MFI) for the pre-oligodendrocyte marker CNPase and the percentage of CNPase positive cells in OPCs exposed to i-EVs or IL4-EVs, or not treated (control). (B) Histograms showing the MFI for the mature oligodendrocyte marker GST-Pi in OPCs exposed to the same conditions. One replicate was analyzed for each condition.

Suppl. Fig.1

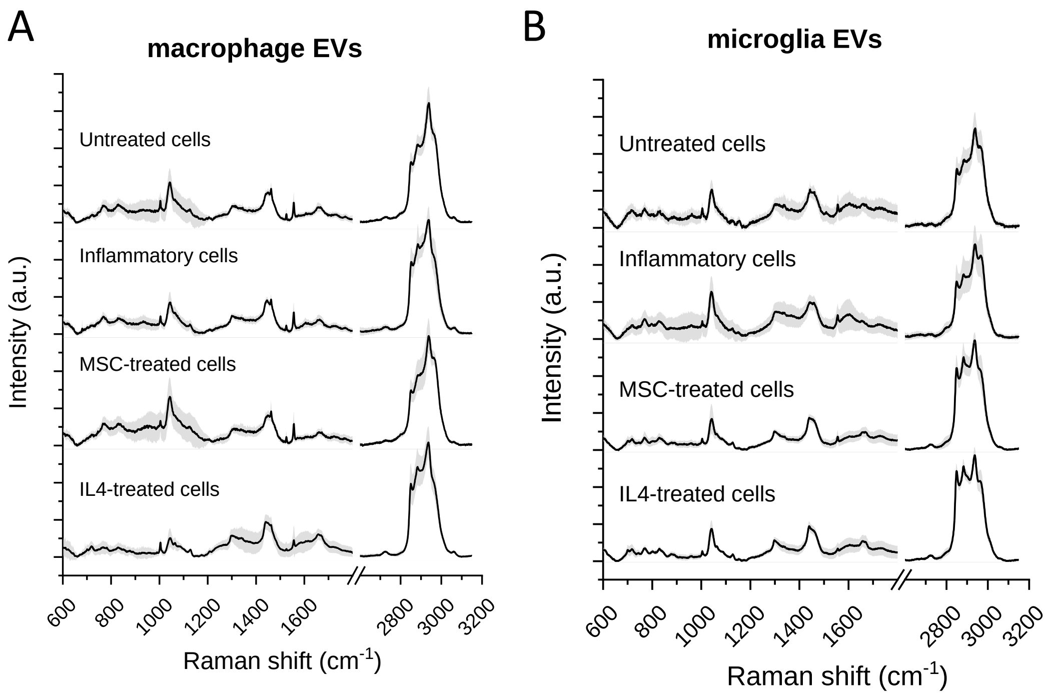

Suppl. Fig 2 Raman spectra of EVs released from macrophages versus microglia
Raman spectra of intact EVs derived from macrophages **(A)** and microglia **(B)** in absence of stimuli (untreated cells) or polarized towards different activation states. Spectra show that the molecular composition of macrophages-derived EVs largely differs from that of microglia-derived EVs and reveal differences and similarities between the four conditions.

Suppl. Fig.2

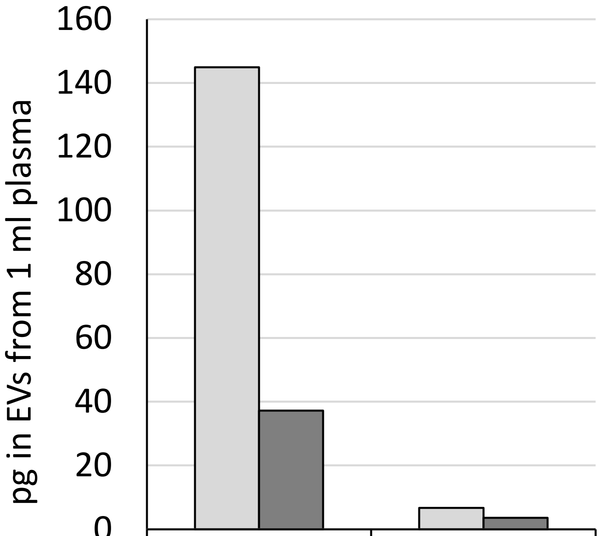

	2-AG	AEA
total EVs	144.96	6.72
IB4+EVs	37.20	3.60

Suppl. Fig.3 2-AG and AEA in plasma EVs
Histograms represent 2-AG and AEA content in total EVs or IB4+ EVs isolated from 1 ml plasma obtained from a pool of three patients affected by relapsing remitting Multiple Sclerosis. pg=picograms

Suppl. Fig.3
